# An architecture for collaboration in systems biology at the age of the Metaverse

**DOI:** 10.1038/s41540-024-00334-8

**Published:** 2024-01-27

**Authors:** Eliott Jacopin, Yuki Sakamoto, Kozo Nishida, Kazunari Kaizu, Koichi Takahashi

**Affiliations:** 1 RIKEN, Center for Biosystems Dynamics Research, 6-2-3 Furuedai, Suita, Osaka 565-0874 Japan; 2 RIKEN, Center for Integrative Medical Sciences, 1-7-22 Suehiro-cho, Tsurumi-ku, Yokohama, Kanagawa 230-0045 Japan; 3 Tokyo University of Agriculture and Technology, Department of Biotechnology and Life Science, 2-24-16 Nakamachi, Koganei, Tokyo 184-8588 Japan

**Keywords:** Software, Software

## Abstract

As the current state of the Metaverse is largely driven by corporate interests, which may not align with scientific goals and values, academia should play a more active role in its development. Here, we present the challenges and solutions for building a Metaverse that supports systems biology research and collaboration. Our solution consists of two components: *Kosmogora*, a server ensuring biological data access, traceability, and integrity in the context of a highly collaborative environment such as a metaverse; and *ECellDive*, a virtual reality application to explore, interact, and build upon the data managed by *Kosmogora*. We illustrate the synergy between the two components by visualizing a metabolic network and its flux balance analysis. We also argue that the Metaverse of systems biology will foster closer communication and cooperation between experimentalists and modelers in the field.

## Introduction

This paper presents the interplay between two systems, *Kosmogora* and *ECellDive*, to support the integration of systems biology research in the Metaverse. It is not easy to define the Metaverse because it is evolving alongside practices^1^ and technologies^2,3^.

We identify at least two trends within the private sector depending on the target usage. The first trend includes industrial stakeholders centered on engineering or manufacturing such as Siemens Digital Industries Software, Bentley Systems, or BMW Group. They define^4^ the Metaverse as real-time, immersive, engineering grade with data integrity and traceability, and collaborative. The second trend includes companies also selling services such as NVIDIA and Meta. They define^4,5^ it as the next iteration of the Internet after text, photo, and videos. This second trend emphasizes the experience of the Metaverse in a broader sense including entertainment^4^, socializing^5^, and work^4,5^. However, stakeholders of the first trend do acknowledge the synergy between the two: “It’s quite important that [the Metaverse] is as easy to use for an industrial application and it’s as much fun as playing a game”^4^.

A qualitative meta-synthesis of various academic definitions of the Metaverse identified^6^ a set of 14 “dominant terms” at the top of which is “immersive”. From this lexicon, we notice that the concerns about data integrity and traceability from the first trend of the private sector do not seem to be much discussed by academics. On the contrary, the lexicon is more in line with the second trend.

Academia mainly relates immersion^2,3,7,8^ in the context of the Metaverse to users embodying avatars^1,8^ in a virtual world. Users are given a virtual representation of themselves that follows their gestures. This, in turn, powers social interactions and collaboration by giving multiple users a shared sense of space, presence and time^9^. In particular, embodied real-time interactions give rise to non-verbal communication via gestures^1,9^ (e.g., pointing at an object). Collaboration in the Metaverse is thus considered real-time or, at least, with little latency. Immersion is reinforced by persistency which is the property of a virtual world to not reset when users log out. Hence, users that connect back in the Metaverse will find it identical to when they left (unless other users modified it). Immersion in the Metaverse is also supported by extended reality (XR) technologies. XR includes virtual reality (VR), augmented reality (AR), and mixed reality (MR), each powered by devices that blend^10^ the real and virtual worlds to produce various degrees of immersive experiences. The second trend of the private sector advertises the Metaverse through XR technologies. But they are separate concepts and we cannot assimilate one to the other^1^. Not every XR application leads to the Metaverse and the Metaverse can exist without XR. The latter is an obligation toward people who do not want to, or cannot, use XR technologies.

Despite its protean definition, there is a consensus that “the Metaverse”, which we speak of in the same way as we say, “the Internet”, is still only a future construct. The tools claiming to abide by the Metaverse paradigm in engineering^4,11^, healthcare^12–14^, or education^15,16^ are only approximations of what the Metaverse will be, and many challenges remain^1,2,7,17,18^. Key issues specific to the Metaverse of biology include the complexity and diversity of biological systems and data, the accuracy and reliability of biological models and simulations, the integration and annotation of biological knowledge and resources, and the engagement and participation of biological researchers and stakeholders.

There has been a surge of specialized XR applications for biology in the last 5 years^19–27^ which now motivate questions about their integration within the Metaverse^28,29^. In this paper, we focus on issues related to biological data and knowledge management to build the Metaverse of biology. For research activity, how do we ensure remote accessibility to biological knowledge, data traceability, and integrity? Once we know how to manage data, how do we project it to users to guarantee a clear mental model? We addressed these questions by separating the issues of biological data access and management from the issues of visualization and interaction. We developed *Kosmogora* to solve the former and *ECellDive* to solve the latter. *Kosmogora* is a server system that communicates with existing biological databases to centralize access to biological knowledge, while *ECellDive* is a VR application based on “dive scenes” to navigate biological knowledge.

## Results

### Centralized management of biological data and knowledge

Data and knowledge in biology are spread across many databases^30–35^. The initial goal of these databases was to enhance asynchronous collaboration between biologists, but they have each grown significantly in complexity. Eventually, we have reached a state where specific knowledge about a database is required for its efficient exploration. Therefore, collaboration in systems biology could now benefit from efforts toward centralization rather than distribution. The metaverse concept is suitable for this task because it is founded on the integration of heterogeneous data from external and internal sources to build coherent environments for users. In our architecture, the links between biological knowledge established outside and the data generated inside *ECellDive* are managed by *Kosmogora*.

There has been a long-term community effort to enable cross-referencing of databases in biology^36,37^. *Kosmogora* is the intermediate for users in *ECellDive* to query these databases. We organize the centralization in *Kosmogora* via translation tables^38^ of the IDs of databases referencing the same object (compounds, proteins, genes,…). In this study, we established a table to translate between the IDs of the referenced in models of the BiGG database^33^ and the MetaNetX database of metabolic networks^35,39^. ID maps are a flexible data structure allowing to quickly expand the centralization of knowledge to other databases in future.

**Fig. 1:**
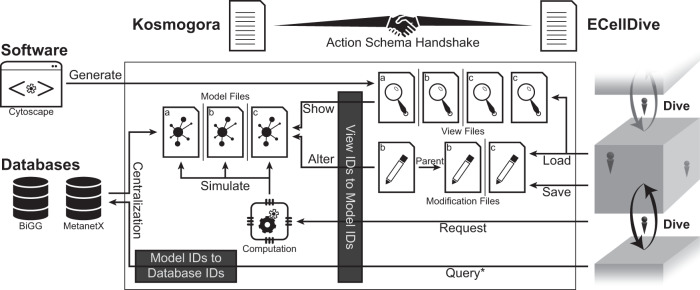
Centralization and management of biological data and knowledge with *Kosmogora*. *Kosmogora*-like servers must implement a list of functions declared by *ECellDive*; the compatibility is checked in the action schema handshake. Kosmogora manipulates three types of files: *model files*, *view files*, and *modification files*. Model files are gathered from online databases and the respective IDs are linked to allow cross referencing. View files are used in *ECellDive* to represent all or a portion of a model file; one model file has at least one view file. View files can be generated by external tools such as Cytoscape^47^. *Modification files* record alterations made by users in *ECellDive* while manipulating the model (e.g., knockout of a reaction in a metabolic network model). *Modification files* may have a parent-child relationship. When a child file is imported into *ECellDive*, all modifications recorded in the parent are also imported. This lineage of *modification files* allows tracing the temporal evolution of a model (“temporal traceability”). *Modification files* are lists of entries describing the modifications and can be compared with each other (“differential traceability”). *As of *Kosmogora* 1.1.X, the queries do not use the databases’ API but process locally downloaded content.

3D immersive environments are often computationally expensive. In our case, we decided to decouple computation resources by processing data and simulations remotely in the instance of *Kosmogora*, while the graphics were solely processed on the standalone VR device (Meta Quest 2) running the instance of *ECellDive*. Data management involved local copies of model files that users are currently working on and that originate from databases. The *model file* is never directly sent to *ECellDive* but is only projected to users via view files which encode the layout of the model in a *dive scene*. Therefore, one *view file* is the interface to one *model file*. A *model file* has, at least, one *view file* (in Fig. 1, model file (a) and (b) have only one matching view file while (c) has two view files). Finally, *Kosmogora* also manages *modification files*, which are records of all users’ actions that alter a *model file*.

The Metaverse promotes interchangeable tools to satisfy one feature in a virtual environment. The combination *Kosmogora+ECellDive* follows this principle, with the former implementing a set of functions to satisfy modules in the latter and not the opposite. Hence, users can connect to any server if it implements the set of hypertext transfer protocol (HTTP) requests required by a module. The application programming interface (API) of a *Kosmogora*-like server is checked by *ECellDive* against its “server action schema” describing the mandatory subsets of commands for each module. If every command in the subset is present in the list of commands implemented by *a* *Kosmogora*-like server, then the module is unlocked in *ECellDive*. Furthermore, if users are connected to multiple *Kosmogora*-like servers implementing the API for a module, then they can choose which to use.

### Tracing the centralized data and knowledge

Traceability is essential in computational biology to discuss the building steps of model with peers. We identified three components in knowledge tracing. First, there is the path of states as a whole—is it possible to identify every intermediate state that led to the current state? We call this “temporal traceability.” In biological knowledge bases, this is manifested by maintaining the possibility of accessing every version of a file. Uniprot^30^ or BioModels^31^ implement such traceability. Second, there is the state itself—how is one state different from another? We call this “differential traceability.” To our knowledge, only Uniprot^30^ provides access to such tracing in biology. Outside biology, *Git*^40^ is a well-known versioning system that enables both temporal and differential traceability. Finally, there is the transition between two states—what were the actions that led to commit to a new state? Here we do not focus on the analysis *a posteriori* of the choices^41,42^ but rather on their manifestation during real-time collaboration. We call this “real-time traceability.” To our knowledge, no biology databases provide such information. This is to be expected, as online databases are “static” environments where knowledge is pushed to (and pulled from) but never created. Text editing platforms, such as Google Documents, are examples that enable real-time traceability.

In our work, temporal and differential traceability are enabled in *Kosmogora* while real-time traceability is enabled in *ECellDive*. *Kosmogora* uses *modification files* to alter model files; a model file can exist with zero or more *modification files* (in Fig. 1, model (a) has no associated *modification files*, model (b) has two, and (c) has one). A *modification file* is a list of modification entries containing information about the authors, the date of the modification, and they can also include a reference to a parent modification file (in Fig. 1, *modification files* of model (b) have such a relationship). Wherever a parent is referenced, the new *modification file* appends all the modifications stored in the parent file upon loading in *ECellDive*. Authorship, date, and lineage of the file all leverage temporal traceability. In addition, as modifications are stored in a list, it is easy to compare multiple *modification files*; thus, leveraging differential traceability.

*Modification files* also have other advantages. First, they only record changes and are not copies of the original file, and so can save storage space whenever the original file is large. This point is essential to address sustainability issues of metaverses. Second, as the *modification files* are stored on the server and not locally on one user’s device, anyone can asynchronously access and further build upon the modifications or backtrack at any time. Third, manipulating *modification files* decouples the actions of the user from the original file, which preserves its integrity. Finally, it is possible to apply multiple *modification files*. This last point is similar to layers in image editing software; a *modification file* is a layer that is applied onto the original data and layers can be combined thus allowing mixing of ideas when testing variations of a model.

### Architecture of centralized data and knowledge

Current methodologies to project data to biologists (e.g., flat lists of proteins^30^, metabolite pathways or genomes^34,43^, rule-based modeling^44–46^ or network^47^ editors, web-based BLAST interface^48,49^) are too narrow and cannot be scaled up with centralization. Next, we present a style of virtual world architecture (in the sense of the architecture of buildings or cities, not software or systems) to organize centralized data and knowledge enabling such upscaling.

**Fig. 2:**
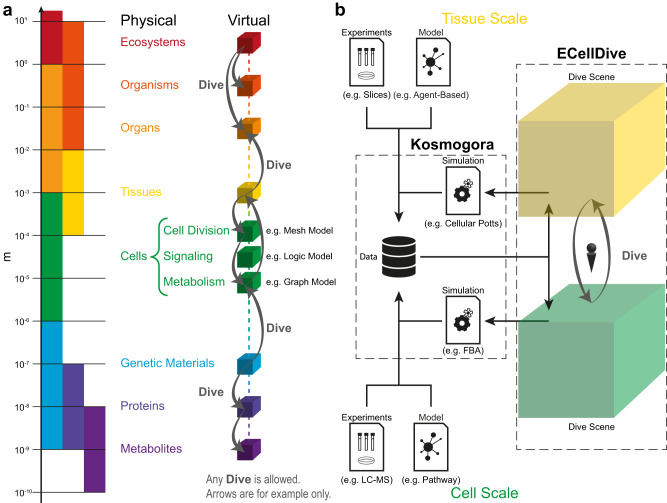
Dive-based virtual world architecture. **a** Using the scale hierarchy of biological subfields for the mental model of the architecture of data and knowledge in a metaverse of systems biology. This aims to help the user understand where they stand in the metaverse of biology similarly to a world map. **b** Zoom of the relationship between data centralization and simulation in *Kosmogora* and its access in ECellDive in conjunction with the user’s movement between dive scenes. The data imported into the dive scenes make them fit either the tissue or cell scale.

Biology is a hierarchical science with subfields targeting the different scales of life. Arguably, ecosystems lie at the top (order of meters), while metabolites are at the bottom (order of nanometers). In *ECellDive*, we suggest following this hierarchy to navigate biological data and knowledge. We project the physical targets of biology to virtual levels that we call “dive scenes.” This does not match the way data are stored within *Kosmogora* or *ECellDive* but rather, dive scenes are concepts to help biologists build a mental model of the biological landscape they are exploring (see Fig. 2). There can be as many dive scenes as needed, and they are not preset for ecosystems or metabolites; a dive scene becomes what users import in it. In Fig. 2a, shows three examples of phenomena at the scales of cellular biology (cell division, signaling, metabolism), which could be attributed to three independent dive scenes to separate modeling approaches (3D mesh model, logic model, and network model).

We call the action of moving from one dive scene to another a “dive”. This allows for a swift change of scales to facilitate knowledge connections between the different subfields of biology. Users can then access knowledge outside of their area of expertise. This is illustrated in Fig. 2b where a user is moving from a dive scene containing data and a model about a tissue, to another dive scene containing data and a model about a cell. All data transit through *Kosmogora* and can originate from experiments or simulation results hosted on online databases.

We implemented a “Dive Travel Map” in *ECellDive* to display the dive scenes a user has visited, and in which order much like in Fig. 2a. This travel map can help users build and maintain their mental model of the biological knowledge they are exploring (see Supplementary Fig. 1).

Research and biology rely extensively on collaboration and interactions between scientists. Currently, real-time collaboration is restricted to in-person or online meetings where collaborators make presentations, perform demonstrations, share results, etc., resulting in most participants passively watching or listening to a few active participants (potentially using some equipment). For example, it is common to have several collaborators looking at a unique PC monitor for in-person meetings, or to be constrained by the content that one collaborator is sharing via screen streaming for online meetings. *ECellDive*, however, benefits from VR immersion to give all collaborators the opportunity to adopt any point of view while still retaining a sense of presence of the other collaborators due to the use of avatars. Moreover, *ECellDive*’s real-time immersive collaboration enables real-time traceability. Compared to temporal traceability, data is not stored on *Kosmogora* or *ECellDive*. Rather, real-time traceability arises as soon as two or more users are following each other’s actions and is contingent to mutual awareness^50^. It follows from their discussions, actions, and decisions about the data. In *ECellDive*, real-time traceability is made possible by the fact that a user can see others’ movements; all users can see others interact with data modules; all users can see others make groups of modules.

### Tangible 3D objects to interact with biological data and knowledge

**Fig. 3:**
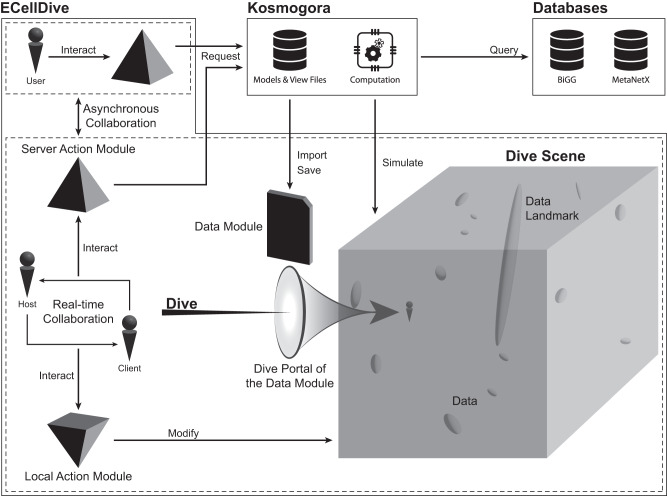
3D objects-based interactions in Dive scenes. Data files, portals, data, interactions with the server, and interactions with data are based on tangible 3D objects in *ECellDive*. In contrast to windows on a screen, 3D objects in the virtual world of a dive scene participate in the feeling of immersion. Those can also appear as “landmarks” in the 3D world. That is, data in the dive scene are physically more prominent than the rest and can be clearly identifiable by users similar to a mountain on the horizon. 3D objects in dive scenes are persistent like objects in the real world. They are means of indirect communication between users in the same work session. For example, a persistent dive portal suggests a user went to visit another dive scene from this one; i.e., it is a knowledge link.

Here, we discuss how we organize and interact with data in dive scenes. First, the virtual environments of metaverses are infinite: regardless of the size of the real-world room the user is in, they can cross kilometers virtually without moving physically. Although the “infiniteness” is similar to panning a 2D canvas on a monitor, the “infiniteness” of a VR environment is more versatile. For example, users are not constrained to a third-person view when looking at data encapsulated in a plot anymore. Instead, data can be mapped in the whole virtual environments to build a landscape users can explore in first-person view. In a “landscape of data” extrema would appear as “landmarks” similar to mountains on the horizon (see Fig. 3). In addition, virtual worlds in metaverses are real-time environments and animations are customary features to help users, or to add contextual information in the virtual world. Continuing with the metaphor of a landscape, exploration can be tedious without a map or a compass. In the case of a “landscape of data” animated virtual objects (i.e., compass) would guide users toward noteworthy regions of the landscape. The core concepts for efficient data visualization (e.g., colors, shape, size, layout, …) on a PC monitor are still valid in a virtual world but we gain degrees of freedom due to the infinite space, the third dimension, and the real-time component.

Second, users can act upon the virtual world through virtual 3D objects related to the notion of tangible UI:^51^ objects in reality may modify the virtual world. In VR, tangible UI is approached by associating 3D shapes to functions and haptic feedback in the controllers. 3D objects give a sense of presence, which we believe is better adapted to biologists because they are used to working with physical tools in laboratories. In addition, the 3D objects are persistent in the virtual world, so their presence becomes a medium for indirect communication and collaboration. For example, in *ECellDive*, users dive between dive scenes through portals with an elliptic shape (see Fig. 3). This portal remains in the dive scene after a user has dived through it. Therefore, a portal becomes information for other users that someone has passed through this point and created a knowledge link between this dive scene and another. This added information participates to the real-time traceability.

3D objects in *ECellDive* have two purposes: to encapsulate biological data (data module) and to provide a way for the user to act on the data (action module). The first category of action modules allows users to communicate with *Kosmogora* (Server Action Module in Fig. 3). The second allows to manipulate data modules locally (Local Action Module in Fig. 3) when it does not require extended computation resources.

### Demonstration: a flux balance analysis in ECellDive

**Fig. 4:**
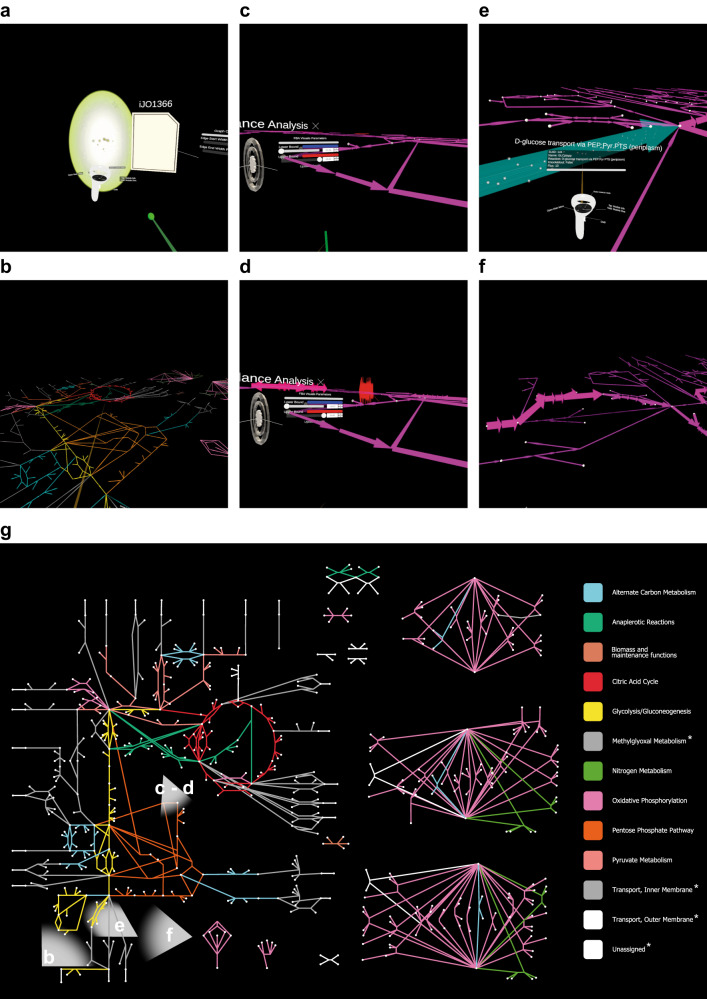
Illustration of the main steps of a flux balance analysis in *ECellDive* on model iJO1366. **a** The portal used to dive into the representation of iJO1366 encoded in the view file. **b** A high view of the pathway after color customization of the edges to group them according to the main subsystems of this metabolism. **c** We performed a Flux Balance Analysis (FBA); the minimal flux value is −45 and the maximal value is +55. Colors interpolate between blue for low values and red for high values. Most of fluxes have value 0 (hence, the pink). **d** Manipulating the width of the edges is much more efficient than relying on colors only. VR immersion enables the concept of “landmark” facilitating the spotting extrema as we would for mountains in nature: the red fluxes are clearly visible. **e** Zoom on a flux; the D-glucose transport through the periplasm. The FBA gave it a value of 10. The white points on the images are animated particles which debit is proportional to the value of the flux. **f** We knocked out the D-glucose transporter and the flux was redirected. **g** An overview of iJO1366’s network made in Cytoscape^47^. This is not available in *ECellDive*. It is provided here only to help locate where the user and its field of view in **a**–**d**. *Colors for the groups were taken to match the situation in **b**, the color conflicts were a choice of the user at the time.

As a demonstration of the dive-based virtual world architecture, real-time collaboration, and the action modules, we used a published model^52,53^ (iJO1366) to perform a flux balance analysis^54–56^ (FBA), a common method in systems biology to analyze the theoretical throughput of metabolic pathways that are in a steady-state. We retrieved the central metabolism of *Escherichia coli* downloaded from Escher^52,53^ and stored it in *Kosmogora*. From there, we imported it into *ECellDive* as a data module and dived into it (see Fig. 4a). In this example, two dive scenes are defined by the root space where the user is dropped when launching *ECellDive*, and the scene defined by the metabolic pathway (see Supplementary Fig. 1).

By default, the metabolic pathway is mapped on a plane. Biologists are familiar with seeing metabolic maps in 2D, and typically newcomers expect the TCA cycle to appear as a circle because that is how it is taught in textbooks. Familiarity with the visualization scheme is an important consideration that must be considered to ease the transition between traditional desktop applications and virtual worlds. Once users are ready, local action modules can customize the layout of the imported biological system.

For example, when the *groupby* action module is added to the *dive scene*, it automatically detects the parts of the model that can be grouped according to their metadata. For a metabolic pathway, the objects suitable for automatic grouping are the metabolites and the reactions. Users can then group edges to highlight functional subsystems of the pathway (e.g., TCA cycle in red in Fig. 4b) and nodes to understand their positions in cellular compartments (e.g., cytosol).

Users can also interact with an action module to perform FBA remotely on *Kosmogora*. After the FBA has been processed by the server, the fluxes values are mapped to the color or width of the edges of the metabolic pathway (see Fig. 4c, d). This is similar to the conventional visualization in applications running on a computer^52,53^, but large flux values are now akin to mountains on the horizon due to the immersion provided by a VR device; this illustrates the concept of “landmarks.”

Users can clamp the width of edges to balance between spotting individual fluxes and global readability. Flux values can also be visualized dynamically due to animated particles (see Fig. 4e), which are instantiated on edges in proportion to each flux value to map the flow rate of particles to the quantity of metabolites involved in the reaction symbolized by an edge. Finally, the numerical value of the flux can be obtained by opening the information panel attached to every edge.

Users can interact with reactions to simulate knockout experiments, which can influence the FBA and reroutes the fluxes (see Fig. 4e, f). Supplementary Figs. 2–5 provide larger in-app captures to follow the chronology from the importation of the view file of a data module to the FBA.

## Discussion

Now, we discuss where our work stands in the current state of the Metaverse paradigm. We have associated *ECellDive* with the metaverse of biology for simplicity, but it is better associated with the metaverse of systems biology. The difference is that a metaverse of biology must cover a wider range of knowledge and practices than a metaverse of systems biology. For example, it is unclear where we would place knowledge about phylogeny, evolution, or mendelism in the dive-based virtual world architecture of *ECellDive*.

Going back to the definitions of the Metaverse^1–9^, we identified seven points relevant for a metaverse of systems biology. First, we have (1) real-time, (2) immersive and (3) multiplatform. There seems to be a consensus on these three between the private sector and academia. This comes from the technology used to leverage metaverses and is quite independent of the systems biology field per se; these three are requirements. Next, as systems biology relies on both experiments and simulations, the data and simulation integrity voiced by some engineering companies is mandatory. A metaverse of systems biology must be (4) engineering-grade (high simulation accuracy, data integrity, and data traceability). We also feel that (5) collaborative and (6) social apply to a scientific metaverse. For (5) collaborative, we mean close relationships among the collaborators during the whole timeframe of the collaboration. The collaborators will likely share data and actively engage in discussions about their projects. In (6) social, we include a broader spectrum of asynchronous (e.g., newsfeeds, articles) and real-time interactions (e.g., talks, posters, conferences), with peers beyond the frame of collaborators as well as with the public. Finally, we add (7) open, which became a major feature of modern science practices via, for example, the Findable, Accessible, Interoperable, Reusable (FAIR) data principles. Therefore, a scientific metaverse should strive to be (7) open from the beginning. Moreover, due to laboratory automation^57–61^ and digital twins of laboratories^62–64^, a scientific metaverse will integrate equipment in the virtual space. It will become possible to run experiments in the real world while interacting with virtual elements in the metaverse. Therefore, we foresee the presence of (7) open equipment in an open scientific metaverse. As an analogy with FAIR data, FAIR equipment could start with a public collection of the virtual counterparts of research institutions (Findable). Visitors could take a virtual tour or watch on-going experiments (Accessible). Visitors could also manipulate the digital twins of equipment provisioned for educational or research purposes (Interoperable). Manipulations could be re-run to verify results of protocols or adapted for different experiments (Reusable).

*ECellDive* fulfills the (1) real-time requirement because it was developed using Unity^65^, which is a game engine dedicated to real-time software (i.e., video games) and (2) immersive requirement because it runs on the Meta Quest 2 VR headset. However, it does not satisfy the (3) multiplatform requirement because it only runs on Meta Quest 2. Developing software for multiple platforms requires a great deal of resources, which will be challenging for any scientific community that wishes to develop a metaverse. This will become progressively easier for XR devices due to standards such as OpenXR but is still a long way off in the future. *ECellDive* provides a way for (4) engineering-grade simulations and data management due to its association with *Kosmogora*. It also demonstrates (5) collaborative features on a host/client architecture. Of course, this architecture does not scale up to the massive multiuser experience that a metaverse aims to be. However, using dedicated servers instead of the host/client architecture would not change the essence of the collaboration scheme of *ECellDive*. The lack of dedicated servers makes it impossible for *ECellDive* to support the professional (6) social life of its users as well as the (7) open stance. Indeed, without dedicated servers, ECellDive does not have the resources to deliver a large social hub where biologists could share the latest news on popular models or discovery trends. Moreover, *Kosmogora* only runs locally which breaks the first two principles of FAIR.

It is difficult to perform a fair comparison of *Kosmogora*+*ECellDive* with current tools that overlap in terms of field (biology), platform (VR), or features (data management, visualization, and simulation), such as Nanome, VR-Omics, Cytoscape, DataHop (see Table 1), and others^19–24^, because these tools focus on a specific aspect of their respective targets whereas our goal with *Kosmogora*+*ECellDive* is to describe an architecture that wraps around these applications and how to navigate between them. In this respect, our work is closer to NVIDIA Omniverse or the in-house pipeline at Volvo (see Table 1) even if the level of invested resources is different.

**Table 1:** Tools overlapping with concepts also present in *Kosmogora*+*ECellDive*.

Tool	Actor	Platform	Field/Subject	Metaverse-Ready^a^	Description
Nanome^24^	Industry	VR	Biology, Chemistry, Pharmaceutics	No	A collaborative tool for molecular design and analysis
VR-Omics^25,62^	Academia	Desktop 2D & 3D + VR	Omics data visualization	No	Desktop to VR pipeline integration for omics data processing and visualization
Molecular Rift^63^	Academia	VR	Biology, Chemistry, Pharmaceutics	No	A tool for molecular structure visualization
Cytoscape^33^	Academia	Desktop 2D & 3D (via plug-in)	Network Data (Biology, Social Sciences, …)	No	Popular tool for display and analysis of network data
Graphia^64^	Academia	Desktop 2D & 3D	Network Data (Biology, Agritech, Social Media, …)	No	Tool for display and analysis of large-scale network data
SpaceTime^65^	Academia	VR	Real-time collaboration	No	Research project for new collaboration schemes and techniques in real-time VR
DataHop^66^	Academia	VR	Data visualization	No	Research project for immersed data visualization where plots are laid out according to the analysis steps of the user
NVIDIA Omniverse^67^	Industry	Desktop & XR	Virtual world creation	Yes	Large development tool suite for engineering-grade digital twins (industrial and scientific) and metaverse applications
Volvo Truck R&D^11^	Industry	Desktop & VR	Automobile engineering R&D	Yes	In-house R&D system for collaborative and immersive design of new trucks

^a^Metaverse-Ready means that the tool includes metaverse constraints directly in its specifications; it does not mean that the tool is a Metaverse or is future-proof against the evolution of the definition of the Metaverse. For example, Nanome and VR-Omics are very good immersive visualizations, but they do not include concepts in their architecture to integrate with the larger scale of a Metaverse. NVIDIA Omniverse and the Volvo Truck R&D were designed to support this larger scale, which makes them “metaverse-ready” in our definition.

It will be possible to develop *ECellDive* such that it supports the requirements of the Metaverse for biology (above the Metaverse of systems biology). However, to ensure that *ECellDive* remains (7) *open*, it will be difficult to rely on a third party to help with the development process. Unfortunately, the skillset required to build a metaverse does not match those required in scientific fields such as biology, and not even computational biology. There is a global increase of programming knowledge among experimental scientists to perform sound statistical analysis, but this is unrelated to the knowhow of software development for a metaverse. For example, further development of *ECellDive* requires:

i. Knowledge of C#, which is hardly taught to students majoring in biology in comparison to Python and R.
ii. Knowledge of the game engine Unity^65^ and its components related to XR devices to produce real-time immersive environments.
iii. Knowledge of software for 3D modeling, visual effects, and textures as well as artistic sense to produce valuable assets.
iv. Knowledge of network programming for online collaboration and socialization.

Requirements (i) and (ii) are lower on other platforms than Unity—the Unreal Editor for Fortnite^66^ and the Verse Language^67^ or Roblox^68^ are trying to ease development of metaverse-oriented applications. However, these platforms are tailored for games, and it is unclear when these more user-friendly tools will be able to deliver engineering-grade environments such as in NVIDIA Omniverse^69^ without too much coding. Requirement (iii) is technologically within reach with tools relying on large language models (LLMs) to produce 3D assets^70–72^. But the legal uncertainty regarding copyright of the data used for training LLMs may conflict with requirement (7) *open*(-ness).

Aside from these technical difficulties, research software development in academia is known to suffer from management issues. Most packages and software are indeed not maintained after publication due to staff turnover and the difficulty of funding a single long-term research project. This is not acceptable for large projects such as a metaverse.

There is hope, however, that any scientific metaverse will manage to aggregate sufficient community input to ensure a wide user base. For example, in the metaverse of biology, experimentalists will use the same tools and mix with modelers and data visualization specialists. Therefore, the concentration of users in one place will likely guarantee constant interest, and eventually fuel long-term support through national grants, much like large biological database projects^30,31^.

## Methods

### Development of Kosmogora

The metabolic pathway *model file* is an SBML file^73,74^ and the *view file* is a CyJson file^47^. We use SBML to describe models because to avoid implementing a custom library for simulations as many simulation packages can use SBML files as input. Currently, we use *COBRA*^75^ in *Kosmogora* to run simulations.

Our approach for the *view files* is to reuse file formats that are already used by different communities wherever possible. A *view file* can be of any format if it stores enough information to display a model in the VR environment; it tells *ECellDive* where to instantiate the game objects (GOs) that will embody the elements of the model. For example, if a *model file* describes a graph (e.g., metabolic network), then the *view file* requires information about the positions of the nodes and the sources and targets of the edges. A *view* *file* may also include labels and metadata for objects present in the model. However, not enforcing a general format for the *view files* has implications. Indeed, for any new *view file* format added to *Kosmogora*, a matching module must be added in *ECellDive* to correctly parse it.

*Modification files* are YAML files with fields tracking the user saving the file (author), date, root model, parent *modification file* (optional), and a list of modifications. *Modification files* are independent of view files which are only spatial projections for *model files*, the core information is the model. Model views may react to *modification files* only if they both contain entries matching the same element in the *model file*. If a *modification file* contains modifications about parts of a model that are currently not in the *view file*, they will still be accounted for in simulations.

Conflicts between *modification files* are avoided by always giving priority to the last file loaded. Indeed, we consider that any modification in the $n$-${th}$ loaded file and targeting an element $e$ of a model will override any modification that also targets $e$ and that has been loaded in one of the $n-1$ previous files. Similarly, when users build genealogies of *modification files* with the parent—child system, the parent is always the last file loaded. Hence, any modification in the $n-1$ previous *modification files* that are not shared with the $n$-${th}$ file is considered “new” and will be explicitly recorded when saving the $(n+1)$-${th}$ modification file.

*Kosmogora* is implemented in Python and utilizes the *Uvicorn* package^76^, which uses HTTP. Therefore, every module in *ECellDive* that interacts with *Kosmogora* (i.e., for importing, saving, and simulating) uses an API to build uniform resource locators (URLs) for HTTP requests. The basic structure of the URLs contains the IP address and the port to reach *Kosmogora* separated by a colon; then a page of the URL is the name of the query to run in *Kosmogora*; and finally, the last page describes the parameters of the query (if any). In *ECellDive v0.11.X-alpha*, users can ask to see the list of models and view files, to import view files, to see the list of *modification files*, to save new *modification files* and to solve the FBA of a metabolic network model.

*Kosmogora* does not communicate directly with remote databases when it needs to retrieve specific information upon a user’s query. Rather, to avoid the layer of complexity added by each database’s specific request API, we locally added target database files in *Kosmogora* (i.e., copying content). This was done for the *BiGG*^33^ and *MetaNetX*^35,39^ databases. Of course, this will not scale up for future applications, but it does not change the purpose of *Kosmogora* in the context of this work, and we will update it later.

### Development of ECellDive

In the following we summarize information about *ECellDive*’s development. More details are available in the documentation for development online (https://github.com/ecell/ECell_Dive/api/ECellDive.html).

*ECellDive* was developed with Unity. It is a professional game engine^65^ originally built to develop video games on multiple platforms (PCs, consoles, XR devices). However, it is now also used in many other fields including architecture, automobile manufacture, cinema, robotics^77^, and AI^78–80^.

A game engine is built around a loop usually called a game loop, which creates the illusion of a real-time virtual environment. Game objects are instantiated, live, and die in Unity’s virtual environment. The behavior of a GO is defined by the combination of components attached to it. Unity has prebuilt components to cover classic aspects of a game such as user inputs, physics, graphics, etc. However, programmers can also implement custom components using C# scripts. A component is created by defining a class deriving from MonoBehaviour that is part of Unity’s code base. We wrote dozens of custom components for *ECellDive* (refer to the online documentation for a full description). Finally, GOs can have parent—child relationships to further increase the complexity of the behavior of a parent GO.

The data and action modules in *ECellDive* are GOs to which we added components to customize their behavior accordingly. Some of the components are part of Unity’s XR package, while others are custom-written C# scripts. These scripts implement features related to the user interface (e.g., highlight, grab, move) as well as the core features of the modules.

We wrote two C# classes to facilitate the implementation of new modules. The first is *Module*, the second is *GameNetModule*. The former is used when a module should only be visible by the user who added it, the latter is used to share the module over the multiplayer network. For example, the server action modules in *ECellDive* (e.g., to communicate with *Kosmogora*) inherit from *Module* because none of them require simultaneous access or interaction. Conversely, a data module is shared among all users, so it inherits from *GameNetModule*.

The metabolic pathway data module implemented for this paper uses the default layout of the metabolic network based on the (X, Y) coordinates of the nodes (i.e., metabolites) stored in a CyJson file inherited from the desktop software *Cytoscape*^47^. Since every user must be able to dive into this CyJson data, the *CyJsonModule* inherits from *GameNetModule*.

Users interact with modules or navigate in the virtual environment by pressing buttons of the controllers. Unity provides components (e.g., *XRActionBasedController*, *XRRayInteractor*, *XRRayInteractable*, …) as part of its XR toolkit to process data from the controllers (position, rotation, and buttons) and make objects in the scene react to the controllers. We reused those components to define three sets of actions to enable users to interact and move in the virtual environment. We also implemented a component (*InputModeManager*) to write the logic controlling how to switch from one set of action to another. *ECellDive* contains a label system informing users of the effect of each button on their controllers for every set of action. The interaction system in *ECellDive* was designed a few months before Meta Quest headset supported *OpenXR* standard (last quarter of 2021) so *ECellDive*’s is only compatible with Meta’s headset as of *ECellDive* version *0.11.7-alpha*.

We used Unity’s package *Netcode for GameObjects*^81^ to implement a host/client architecture in which a user, the host, runs the server that will synchronize the virtual world for all other connected users (the clients). This network solution does not require additional hardware because the instance of the server runs directly on the device of the host. However, as there is no specialized hardware, the number of synchronized clients is limited by the computational power and network bandwidth of the host. *ECellDive* runs on Meta Quest 2 and it is recommended to not exceed four users to avoid latency and ensure a good experience in a dive scene. In this framework, a user creates an instance of a collaboration session at a specific IP address, and clients (collaborators) can join the session. In a work session, clients can import, edit, and save data with the link between *Kosmogora* and *ECellDive*. Future metaverses will have a much larger network infrastructure than *ECellDive* with dedicated servers to oversee the real-time collaboration of more than four users. For example, current multiplayer video games can manage real-time lobbies of several dozens of users without latency issues. In single-player mode, the user is their own host, while in multiplayer mode, a player can host others over a Local Area Network (LAN). Connection over the internet with the host/client architecture is theoretically possible but requires every host to configure port forwarding on their router. This can prove complicated if the host is within the network of a laboratory where the router’s configuration is probably under strict management.

**Fig. 5:**
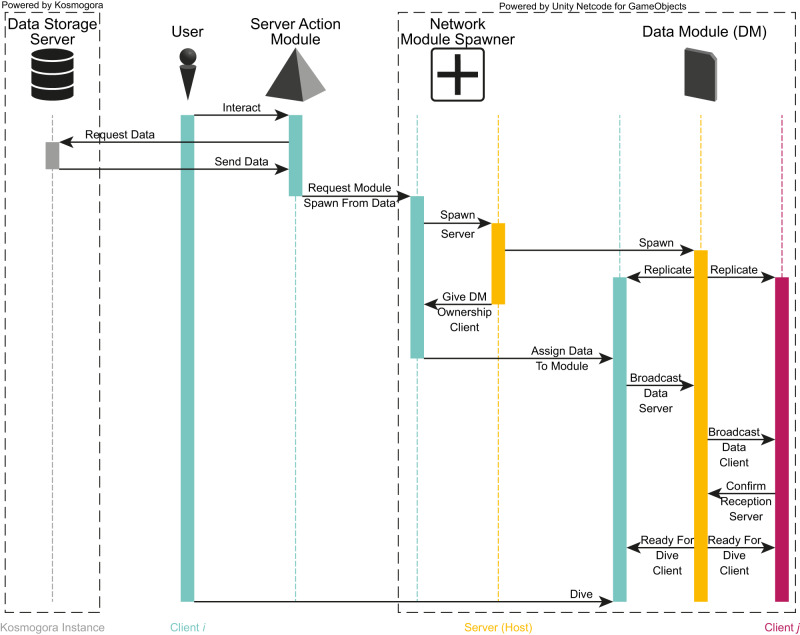
Simplified sequence diagram for data importing in the Client/Host work session of ECellDive. A Client in the work session interacts with a server action module targeting a data storage to request for data. Once the data is received, it triggers a cascade of calls in the work session to create a 3D object encapsulating the data (data module) for all clients in the session. After every client confirms that it received all the data, any client can dive into the data module to visualize its content and further interact with it.

Connection of a client to a host is protected by a password set by the host. When a client connects, he will exchange data with the server (the host) to synchronize the state of its *dive scene*. This operation is simplified to some extent thanks to the API of Netcode for GameObject but we encountered difficulties when sharing large data files (e.g., models and views). Indeed, Netcode for GameObjects was not designed with this use case in mind. Rather, it is meant to support multiplayer games where smaller messages between the server and the clients. This is an issue in our case because we need to synchronize large data files: when a client decides to import a module, the content of that module must be shared to all other clients. The current sharing sequence between users is depicted in Fig. 5. A client contacts *Kosmogora* through a Server Action Module (left half of the figure); then, when the client receives the data, it is automatically forwarded to the server that will broadcast it to the other users connected to the session (right half of the figure). When the data is large, the “Broadcast Data Server RPC” handles the partitioning into smaller chunks and sends one per frame. Then, the chunks are re-assembled on the side of the receiving clients.

### Reporting summary

Further information on research design is available in the Nature Research Reporting Summary linked to this article.

### Supplementary information

Supplementary figures

Reporting Summary

## Data Availability

No additional data was produced for this study. This study used a model of the metabolism of *Escherichia coli* str. K-12 substr. MG1655 published by other authors under ID “iJO1366” in *BiGG Models* database (http://bigg.ucsd.edu/models/iJO1366) for demonstration of the combination *Kosmogora*+*ECellDive*.

## Code availability

*ECellDive* is available on Github (https://github.com/ecell/ECell_Dive) under MIT license. *Kosmogora* is available on GitHub (https://github.com/ecell/kosmogora) under MIT license*. Id2Id* (Translation Tables) is available on GitHub (https://github.com/ecell/id2id/tree/main) under MIT license.
