# Supplementary material for: An architecture for collaboration in systems biology at the age of the Metaverse
Source: NPJ Syst Biol Appl. 2024 Jan 27;10:12. doi: 10.1038/s41540-024-00334-8 (PMC10821884; doi:10.1038/s41540-024-00334-8)
Supplement: Supplementary file 1 — Supplementary figures [file 41540_2024_334_MOESM1_ESM.pdf]

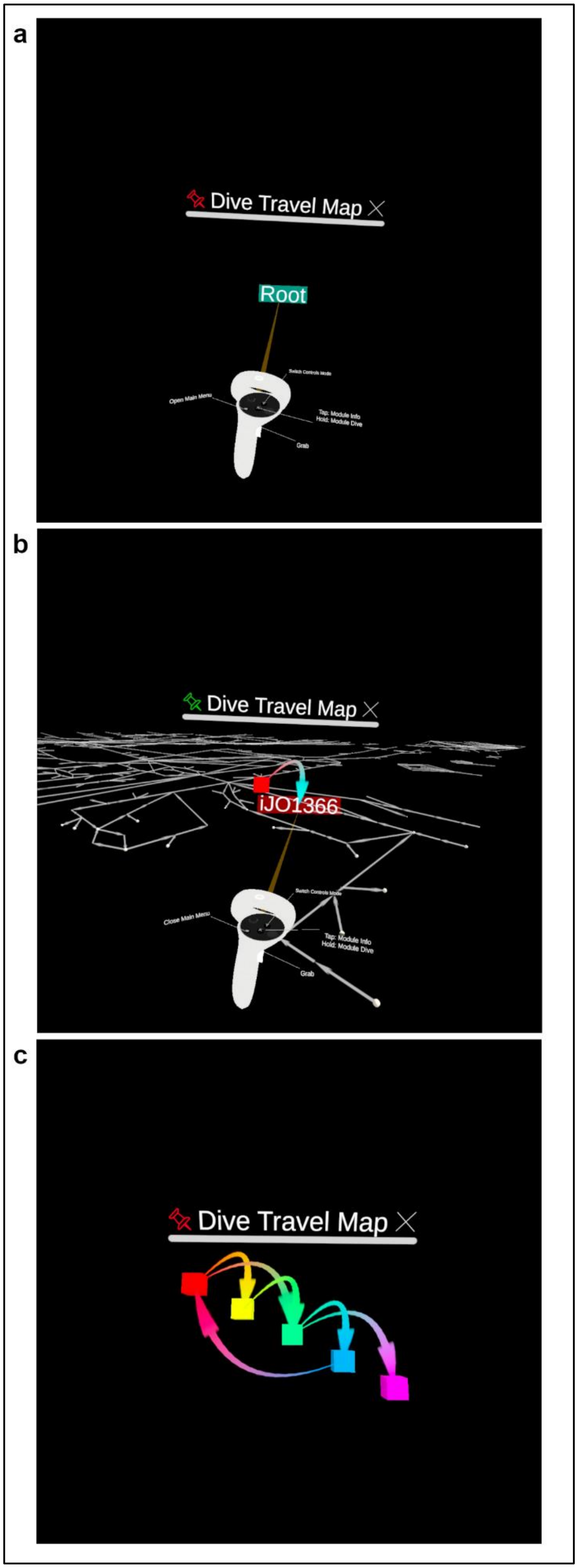

Supplementary Figure 1: Illustration of the "Dive Travel Map" in ECellDive. Dive scenes are represented as colored cubic nodes in a graph. Users can point at the node to transition to a highlighted mode where the name of the dive scene appears within the cubic node and the color switches to the invert of the default color. a) The initial dive travel map when the user has not moved anywhere from the root dive scene. b) The dive map shows that the user loaded the data module of iJO1366 in the root scene and dived into it. c) An example of a more complicated Dive Travel Map. The depth of the dive follows the HUE gradient and is organized from left to right. Arrows are colored by blending the color of their dive scene of departure and arrival.

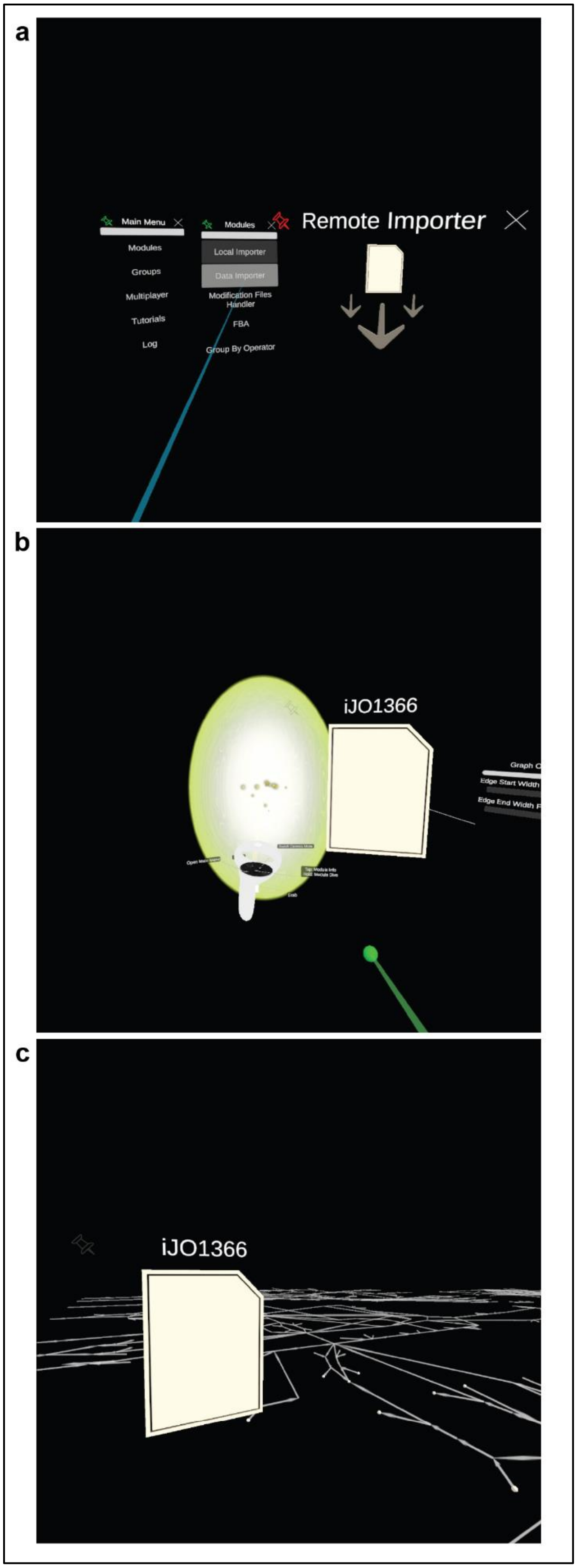

Supplementary Figure 2: Import and dive into the data module iJO1366. a) The server action module to import the view file from Kosmogora. b) The portal used to dive into the representation of iJO1366 encoded in the view file. c) The view when arriving in the dive scene.

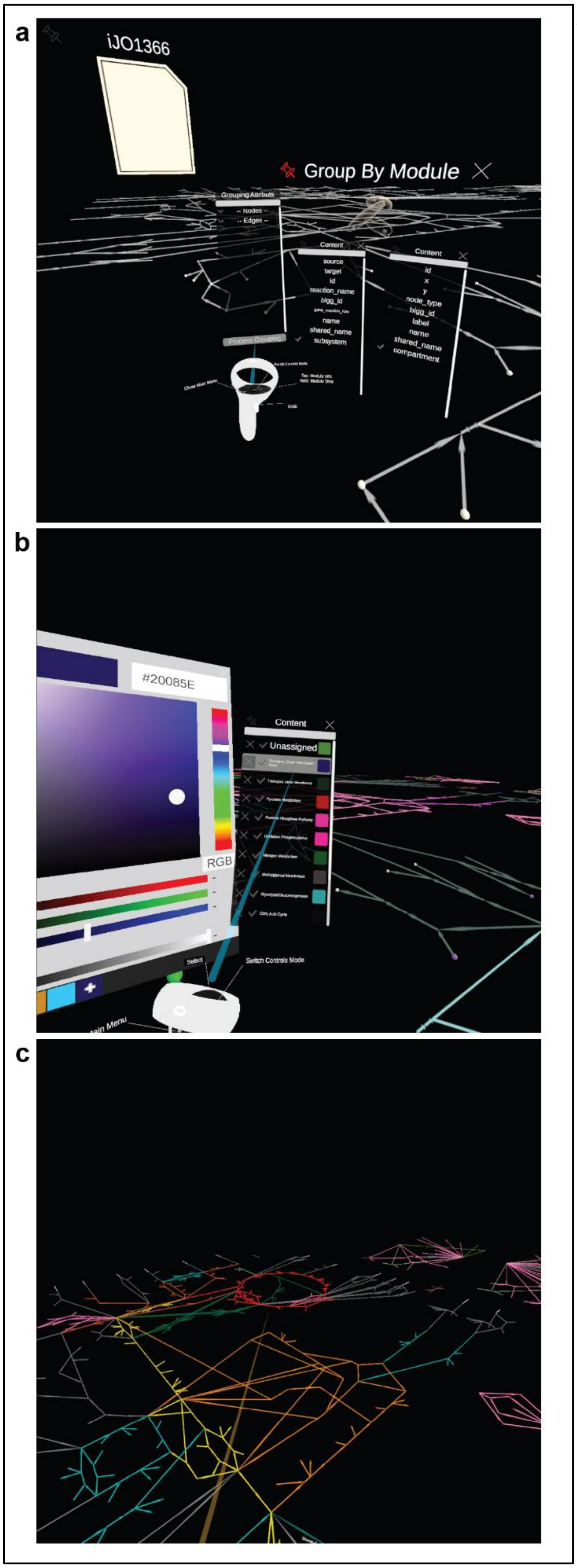

Supplementary Figure 3: GroupBy action module to identify groups of edges or nodes according to their metadata. a) The GroupBy module and its option menus. b) The color picker menu to customize the color of each group. c) A high view of iJO1366 with the edges colorized.

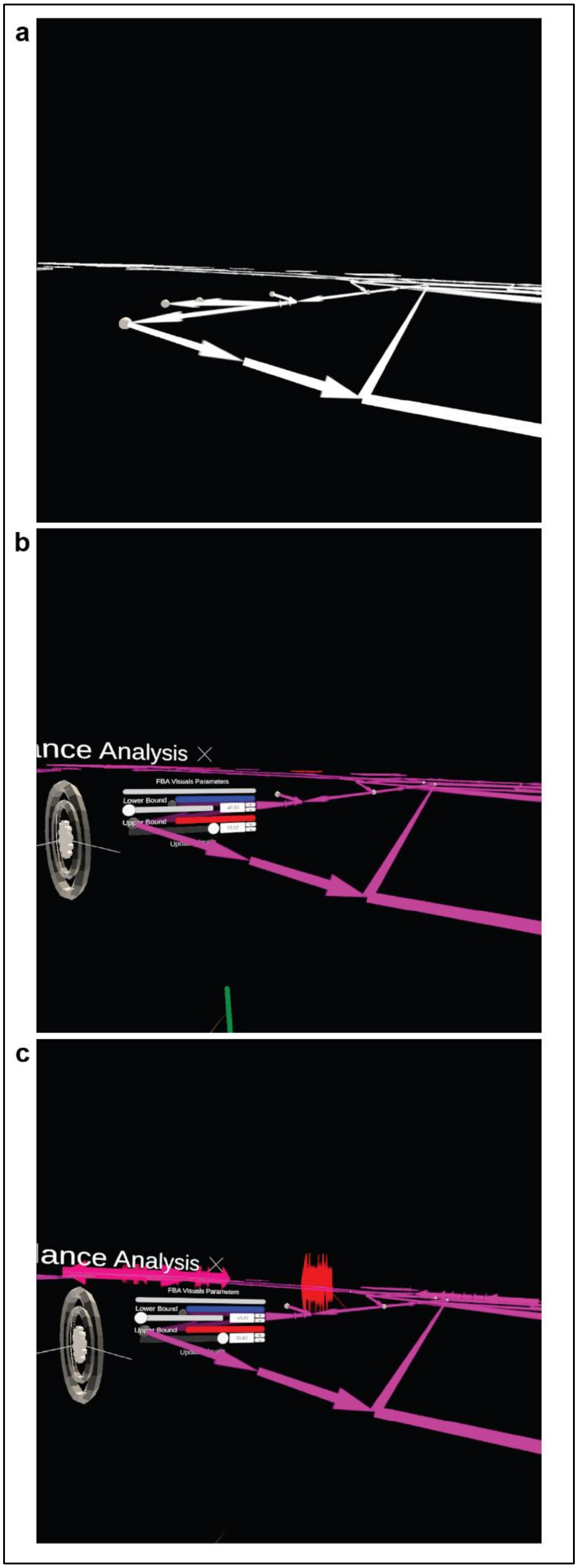

Supplementary Figure 4: Flux balance analysis (FBA) and the concept of "landmark". a) The default visuals of the pathway. b) Performed an FBA and mapped the fluxes' values to colors: blue for low values and red for high values. Most of fluxes have value 0 (hence, the pink). Lowest value is -45 and the highest is +55. c) Mapping the fluxes' values to the width of the edges is a better choice. This is also possible in non-VR apps, but VR immersion allows to better spot the extrema: they are "landmarks" similar to mountains in natural landscapes.

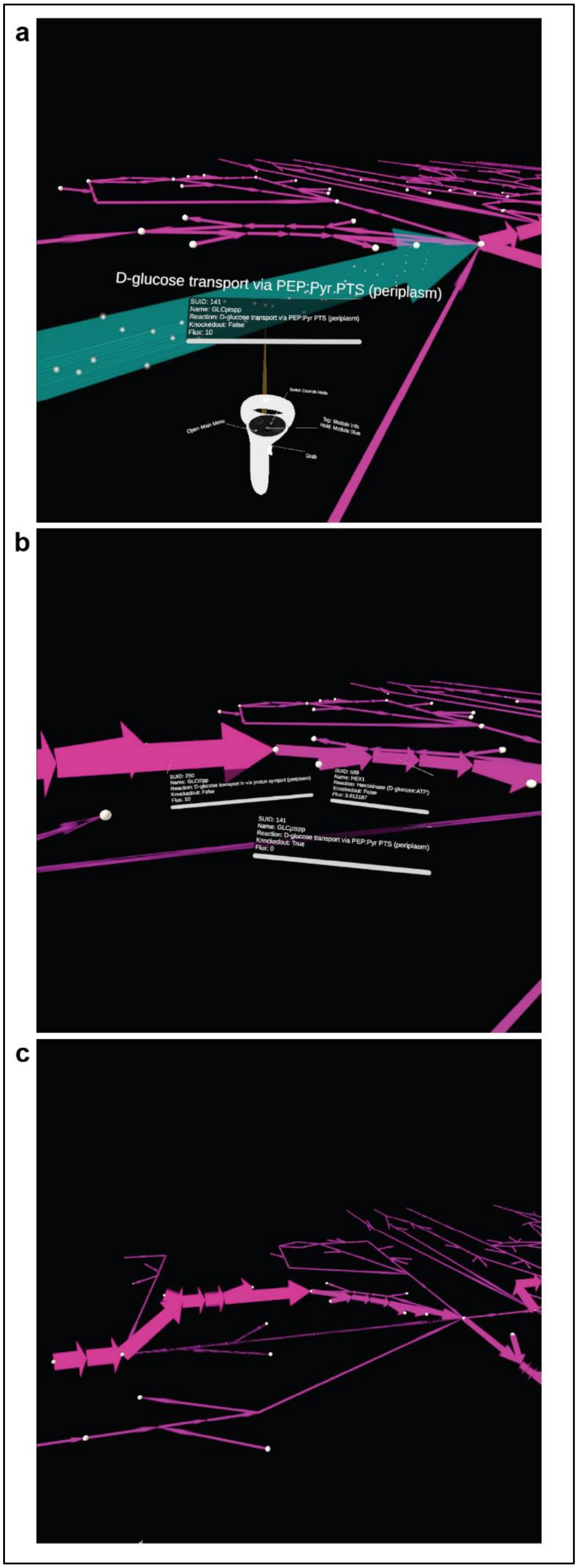

Supplementary Figure 5: Flux balance analysis (FBA) and knockout of a reaction. a) Zoom on a flux; the D-glucose transport through the periplasm. The FBA gave it a value of 10. The white points on the images are animated particles which debit is proportional to the value of the flux. b) We knocked out the D-glucose transport via PEP Pyr PTS (new flux value is 0 as expected), and the glucose import was rerouted through the D-glucose transport via proton symport (new flux is 10, completely taking over the knocked-out transporter). c) A view from farther away to better visualize the rerouting.
